# Quetiapine-Related Deaths: In Search of a Surrogate Endpoint

**DOI:** 10.3390/toxics12010037

**Published:** 2024-01-03

**Authors:** Ivan Šoša

**Affiliations:** Department of Anatomy, Faculty of Medicine, University of Rijeka, 51000 Rijeka, Croatia; ivan.sosa@uniri.hr

**Keywords:** forensic toxicology, quetiapine, relevant matrix, tissue modeling

## Abstract

Quetiapine is a second-generation antipsychotic drug available for two and half decades. Due to increased misuse, prescription outside the approved indications, and availability on the black market, it is being encountered in medicolegal autopsies more frequently. For instance, it has been linked to increased mortality rates, most likely due to its adverse effects on the cardiovascular system. Its pharmacokinetic features and significant postmortem redistribution challenge traditional sampling in forensic toxicology. Therefore, a systematic literature review was performed, inclusive of PubMed, the Web of Science—core collection, and the Scopus databases; articles were screened for the terms “quetiapine”, “death”, and “autopsy” to reevaluate each matrix used as a surrogate endpoint in the forensic toxicology of quetiapine-related deaths. Ultimately, this review considers the results of five studies that were well presented (more than two matrices, data available for all analyses, for instance). The highest quetiapine concentrations were usually measured in the liver tissue. As interpreted by their authors, the results of the considered studies showed a strong correlation between some matrices, but, unfortunately, the studies presented models with poor goodness of fit. The distribution of quetiapine in distinct body compartments/tissues showed no statistically significant relationship with the length of the postmortem interval. Furthermore, this study did not confirm the anecdotal correlation of peripheral blood concentrations with skeletal muscle concentrations. Otherwise, there was no consistency regarding selecting an endpoint for analysis.

## 1. Introduction

Quetiapine is an atypical antipsychotic drug (a second-generation antipsychotic drug) used to treat schizophrenia, bipolar, borderline personality, and major depressive disorders; broadly speaking, this treatment has numerous neurocognitive, neuroprotective, and potential off-label indications [1,2]. Developed in 1985, the US approved quetiapine for medical use in 1997; now, it is on the World Health Organization’s List of Essential Medicines [3,4]. Regarding the non-approved uses of approved drugs, the most frequent such use for quetiapine is its wide use as a sleep aid due to its sedating effects [5,6]. The benefits of off-label use do not appear to outweigh the side effects. Nevertheless, it is reported to treat conditions such as Tourette’s syndrome, musical hallucinations, etc. [7,8,9]. Unlike most other antipsychotics, its hypnotic and sedative effects offset any problems with patient compliance.

Quetiapine’ appears to have low dopamine receptor affinity and intense antihistamine activity, which renders it similar to sedating antihistamines [10]. Approximately 90% of serotonin in the human body is stored in the gastrointestinal tract, and quetiapine has a moderate affinity for its receptors [11]. Notwithstanding, quetiapine shows an affinity for various neurotransmitter receptors [12]. Not only does it enhance the serotoninergic transmission, but serotonin, a key neurotransmitter of the brain–gut axis, also plays a vital role in the pathogenesis of emotional distress and gastrointestinal diseases [13]. Specifically, it binds serotonin (5-hydroxytryptamine; 5HT) 5HT2A, adrenergic (α1), muscarinic, and histaminergic receptors, and it has a relatively weak affinity for dopamine D2 receptors [14,15], with an occupancy half-life about twice as long as that for plasma. All of these are cell-surface receptors that intervene in cellular communication.

For quetiapine toxicity to be fatal, it is necessary to combine it with other drugs [16]. Acute overdose typically results in sedation or hypotension and tachycardia, but cardiac arrhythmias, coma, and death have also been reported [17]. For some of them, severe overdosage may result in seizures requiring intubation/mechanical ventilation.

Some cases are hallmarked by cardiac and sinus tachycardia [18,19,20]. Generally, 10–25 mg/L levels are observed in the blood samples obtained from fatal cases during postmortem examinations. Non-toxic levels in postmortem blood extend to around 0.8 mg/kg, but, at the same time, toxic levels in postmortem blood can begin at 0.35 mg/kg. The serum or plasma of quetiapine overdose survivors had concentrations ranging from 1 to 10 mg/L [21,22,23].

Even though the blockage of histamine-1 receptors produces the soothing effect of quetiapine, arrhythmogenic effects result from the channel inhibition of the ether-a-go-go-related gene (hERG). This may influence the QT interval [24]. The presence of some cardiovascular pathologies, for example, coronary disease, could be the lethal trigger if quetiapine is used, as seen in polydrug intoxications [25,26]. Quetiapine’s deadly effect is governed by whether some medication potentiates this inhibition effect and, if so, to what extent [27,28]. As for respiratory depression, Culebras et al. reported its incidence in three patients on combined antipsychotic–opioid therapy [29]. In randomized clinical trials (RCTs) involving humans, considering the interactions of first-generation antipsychotics and morphine, sedation was scored on a sedation score tool. In eight of the fourteen RCTs, increased sedation scores were reported when morphine and droperidol were combined [19,27]. After the drug’s ingestion and its rapid absorption, it reaches the maximum plasma concentration after 1.5 h, where it binds mostly (83%) to non-specific plasma proteins (human albumin) [13,15,30]. Quetiapine’s bioavailability depends mainly on its first-pass metabolism, which is as poor as 9% [31,32]. Notably, the liver metabolizes many drugs, resulting in the production of water-soluble compounds that can be excreted via the bile [33]. In one stage, this process relies upon the “phase 1 reactions” mediated by cytochrome p450 (CYP). Oxidation, reduction, and hydrolysis reactions are mainly directed by the CYP isozyme CYP3A4. This explains why any drug interaction that modifies quetiapine’s metabolism and pharmacokinetics is more likely to occur with drugs that are inhibitors or inducers of CYP3A4, rather than inhibitors of CYP2D6.

Bearing all of this in mind, this systematic review of the literature aims to identify studies with sufficient laboratory data to identify an alternative matrix to be used in the forensic investigation of quetiapine-related deaths.

## 2. Use and Misuse

In a formal sense, issues related to the misuse and abuse potential of quetiapine have not been regarded as a danger. However, those who administer quetiapine should be cautious when prescribing it to individuals with a history of substance abuse (particularly with opioids or anxiolytics). These individuals are “loose cannons” and are at increased risk.

Typically, people whose deaths are related to quetiapine are men in their mid-forties. Their leading causes of death at this age are drug toxicity and natural diseases. Less frequently, however, these deaths are linked to physical assaults [17].

Occasionally, quetiapine is associated with drug misuse, but it has limited potential for misuse [34]. Misuse is most often seen in patients with a history of polysubstance abuse and/or mental illness (especially those who are detained in prisons or secure psychiatric institutions) because the limited access to alternative intoxicants brings quetiapine to the fore.

However, quetiapine has been found to be associated with drug-seeking behavior more than any other atypical antipsychotic. It has standardized street prices and slang terms, such as “Q-ball” (referring to the intravenous injection of quetiapine mixed with cocaine), either alone or in combination with other drugs [16,19].

### Quetiapine-Related Fatalities and Fatal Toxicity

Due to increased misuse and availability on the black market, quetiapine-associated deaths are frequently seen in medicolegal practice. Unintentional self-poisoning fatalities are classically related to substance abuse, mental health issues, and physical health problems; quetiapine is no exception. Fatalities—complex suicides and suicide attempts involving antipsychotic or sedative–hypnotic medications are frequently seen [35,36,37]. Poisoning homicides are rare, though they have been described, and quetiapine is used only to incapacitate the victim (as in pediatric homicides) [16,38,39].

**Figure 1:**
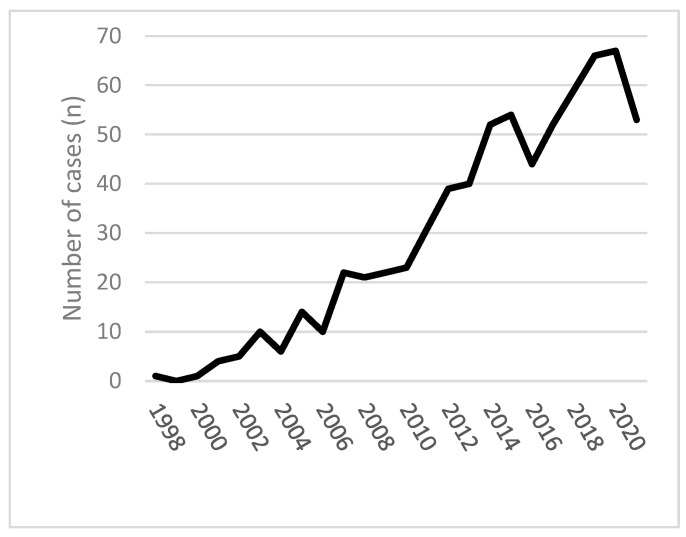
Quetiapine-related mortality in England and Wales 1998–2021 [40].

Some reports on quetiapine-related deaths and series lack clinical details or provide only single quetiapine serum concentrations rather than a kinetic course. However, increasing numbers of studies provide more detailed clinical and analytical data on severe overdose cases. Available data from 1998 to 2021 in England and Wales could be a helpful introduction to the field. In Figure 1, six hundred ninety-six deaths involving quetiapine were presented [40]. A similar, increasing trend of misuse, non-fatal, and fatal overdoses was registered in Victoria (Australia) in the study (2006–2016) conducted by Lee et al. [41]. Mortality data from the European Union are not available

Kales et al. provided estimates of the direct mortality risk over 180 days, comparing individual antipsychotic agents and valproic acid. The mortality risk was found to vary, ranging from quetiapine (lowest) to haloperidol (highest). In fact, quetiapine use had the lowest effect on mortality, with a 3.2% (95% CI = 1.6–4.9%; *p* < 0.01) higher mortality risk than antidepressants (31; 95% CI = 21–62) [42,43].

As reported by Breivik et al., a Norwegian cohort showed no clear relationship for the length of the postmortem interval [44]. Their study showed that the postmortem interval was weakly correlated (positive correlation) with the quetiapine concentration in peripheral blood (mg/L). Moreover, the regression model was invalid (*p* = 0.27) with poor goodness of fit (R2 = 0.16). The same was found for central blood, brain, muscle, and liver tissue. This result agreed with the study of Vignali et al., where a weak positive correlation of postmortem interval was noted only for liver tissue [45]. When the post-mortem interval has been so long that extensively putrefied bodies are assessed, the analysis of entomological samples may support and complement the toxicological results [46]. Even in the case of dried blood spots, the quantification of quetiapine is possible with good recovery rates, within the concentration range of 0.05–1.0 μg/mL [47].

## 3. A Systematic Review Strategy and a Meta-Analysis

**Figure 2:**
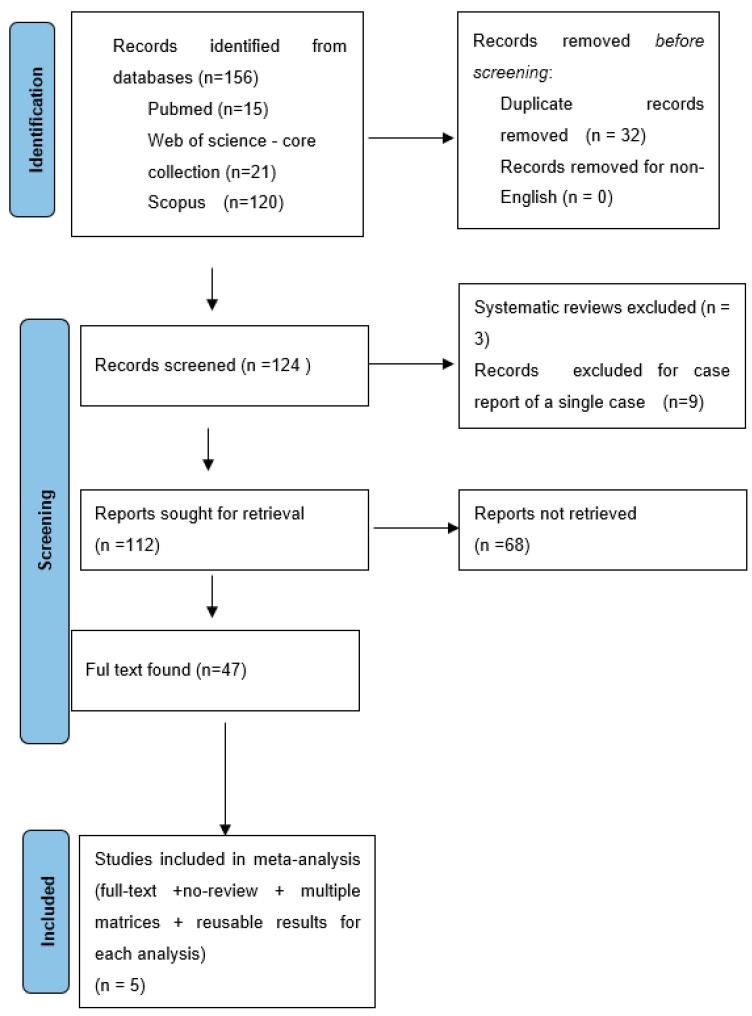
Methodology of literature search—systematic review according to the PRISMA guidelines [49].

This study used a systematic literature review based on PRISMA guidelines to assess the postmortem management of quetiapine-related deaths (Figure 2) [48,49]. The term “quetiapine-related death” refers to deaths where quetiapine was linked to the cause of death anywhere in the causal chain. The literature is abundant in such cases; they are either complex suicides or homicides (where quetiapine was the reason for sedation/incapacitation), accidental intoxications, or polydrug overdoses.

Starting from their inception, PubMed, Web of Science—core collection, and the Scopus databases were screened for “quetiapine” AND “death” AND “autopsy.” After finding 156 records, 32 duplicates were eliminated after being found using the automated bibliography tool. Finally, three systematic reviews, and nine case reports of a single case, were eliminated. Such an approach resulted in 112 entries (English language, “no-single case “case report, original papers, literature reviews, and meta-analyses), of which 47 full texts were available. Five researchers listed in Table 1. involving post-analytical results obtained from multiple matrices were included in the meta-analysis and discussed in this paper (therefore, were inclusion/exclusion criteria).

**Figure 3:**
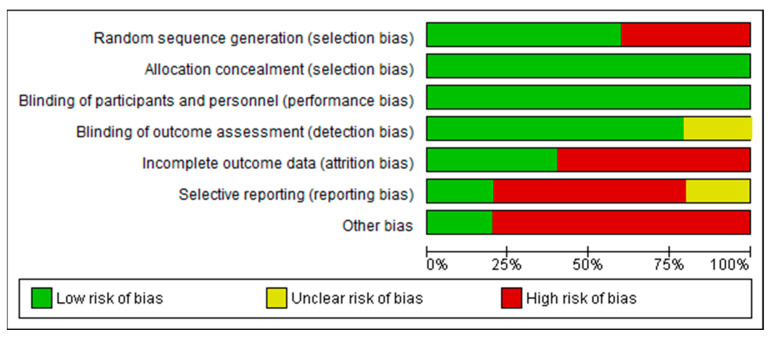
Risk of bias graph for studies included in meta-analysis: review authors’ judgments about each risk of bias item presented as percentages across all included studies.

The meta-analysis correlation of blood quetiapine with other matrices lacks a marked significance level (*p*-value). In cases where this was missing, the correlation could not be proved, so this was considered to be incomplete outcome data (attrition bias, Figure 3). The same was the case in the absence of a marked control cohort. Studies by Anderson and Fritz [50] and Vignali et al. [45] have the form of case reports, even though they do present several cases (Table 1), so this is considered a risk of selection bias (random sequence generation issue). The reason for this problem lies in the practice of forensic pathologists (and all forensic toxicologists are either pathologists or are related closely to forensic pathologists) to rely on experience and individual customary practice in formulating their opinions is a potential source of low goodness of fit or statistically insignificant results in some cases. Conversely, case reports play a critical role in defining new entities, applying toxicological expertise, and obtaining data that other researchers could not accept due to regulations Not considering “post-mortem factors” was considered an “other bias” issue.

## 4. Relevant Matrices

**Figure 4:**
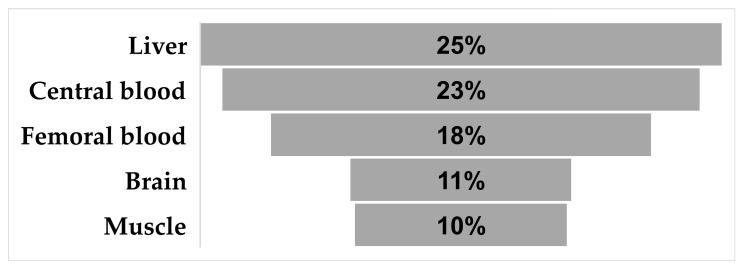
Matrices used in >10% of cases.

Out of five studies included in this meta-analysis, 238 toxicological analyses involving 63 postmortems were performed. Although analyses were performed on a series of different matrices, those that were most frequently used (and are more traditional) are given in Figure 4.

### 4.1. The Liver and Its Lobar Structure

The liver is a highly vascularized, large (typically weighing around 1.5 kg), and encapsulated organ situated to a large extent in the upper right front portion of the abdomen. It is divided into two major lobes; the smaller left lobe partially overlays the ventricle [52,53].

The left lobe is smaller and more flattened than the right. Its undersurface presents a gastric impression and omental tuberosity. Brevik et al. prepared a report of seven participants (7/14, 50%) from whom paired samples of liver tissue were obtained (both lobes). A paired t-test of two samples for means established no significant difference regarding quetiapine accumulation in either lobe. The left liver lobe is most likely more susceptible than the right lobe to the postmortem redistribution of zopiclone, and some of its constituents are thinner due to its anatomical proximity to the stomach (see Table 2) [54,55]. The documented postmortem redistribution of the drug from the biliary system can also contribute to its apparent accumulation in hepatic tissue [44,56,57]. Classic biliary anatomy includes the left hepatic duct, which emerges from the umbilical fissure along the inferior border of the left lobe. The right hepatic duct drains the right liver lobe and comprises two major branches, the right posterior duct and the right anterior duct [58].

### 4.2. First-Pass Effect and the Liver

The liver is the body’s primary site for drug metabolism and contains the largest quantity of the critical cytochrome enzyme system, liver alcohol dehydrogenase, and many other enzymes. Like most xenobiotics, including drugs, quetiapine’s pharmacology and toxicology are largely inextricably linked to its metabolism. Due to its significant metabolic potential, central anatomical position, and ability to take away chemicals from the blood, the liver constitutes an organ with a high susceptibility to the effects of xenobiotics. The liver’s involvement is the most obvious in transaminase elevations. These typically occur by the third week of treatment, and levels return to baseline with continued quetiapine administration [56].

The drug’s volume of distribution while it spreads throughout the body is 10 ± 4 Ukg [15,50]. Quetiapine is orally administered as a fumarate salt in the form of tablets. Daily doses in adults range from 150 to 750 mg, and steady-state concentrations are achieved within two days of dosing [59]. No specific plasma proteins that carry quetiapine were identified; however, it is converted into the active proteins and metabolites norquetiapine and 7-hydroxy quetiapine [60,61]. Both were assessed in patients autopsied in the study of Vignali et al. The authors found (peripheral) blood levels of norquetiapine to be 258.93 (95% CI = −22.63–540.48). Blood levels of 7-hydroxy quetiapine were 45.88 (95% CI = −8.24–83.52) [22,45].

The cytochrome P450 (CYP) system has been observed to extensively metabolize quetiapine in the liver, with less than 5% of the original drug appearing in urine (and minimally in other excretions). Around 73% of 150mg of quetiapine radiolabeled with 100 mCi ^14^C was recovered in the urine and 21% in the feces within 168 h of administration. The mean terminal half-life of quetiapine is about six hours; in its unchanged form, it accounted for less than 1% of the excreted substance [15,62].

Though quetiapine is excreted with urine, it has a low renal elimination rate (less than 5%) and a relatively large volume of distribution (Vd = 10 l/kg), so forced diuresis is no longer recommended [63,64]. The elimination half-life can be easily calculated as follows:

As assessed in several case series, the concentration level of quetiapine was noticeably higher in the liver tissue than in any other postmortem sample [18,44,50]. This paradigm was most evident in the study of Parker and McIntyre (16.09 (CI 95% = 4.96–27.22) mg/kg). However, the linear regression model showed no statistical significance (the *p*-value was 0.09). Anderson and Fritz showed a more distinct and statistically significant positive correlation of quetiapine concentrations between the peripheral blood and liver tissue (R2 = 0.99; *p* = 0.01), although their study consisted of only five participants who were eligible for the linear regression model (27.86 (CI 95% = −31.05 to 86.77 mg/kg)). Another study that assessed quetiapine concentrations in eight liver samples reported 5.11 (CI 95% = 1.11–9.11 mg/kg) [51]. In this regard, the study that included the most sizable cohort was that of Vignali et al. from 2020. It comprised 12 liver samples with mean quetiapine concentrations of 1002.9811 (CI 95% = 57.64–1948.32 mg/kg) [45].

### 4.3. Liver Tissue from Fresh Cadavers

Resected liver biopsies can be sliced with retained original cellular diversity and in vivo cellular architecture. They can be cultured ex vivo for two weeks [65,66]. Routine toxicology is performed on these tissues using mass spectrometry (GC-MS) or specific high-pressure liquid chromatography (HPLC) [30,67]. Both methods are relatively sensitive, with a limit of quantification for HPLC of μg/L, and the GC-MS method is accurate to 2 μg/L [15] (see Table 3).

### 4.4. Liver Tissue Modeling

Normal hepatocytes, constituting nearly 60% of the total cell population within the liver, along with the HepaRG cell line, are capable of performing the majority of liver functions, including many drug-processing activities at various levels [68]. Transcribing liver-specific genes at high levels without fresh human tissue is challenging, but it can even provide differentiated hepatocyte-like HepaRG cells. In fact, it is more successful than any other liver cell line. As HepaRG cells express most of the drug-processing genes, including major CYPs and UGTs, it should not be surprising that these cell lines have been used as pharmacological and toxicological models [68,69,70]. According to Le Daré et al., a higher quetiapine metabolism was observed in differentiated HepaRG cells (50% of quetiapine metabolized) compared to pHH (25% of quetiapine metabolized) [71,72,73]. This helps meet the desired feature of human cell models, stably expressing the functional properties of the in vivo cells they are derived from in order to predict the toxicity of chemicals [74]. Indeed, in vitro models of human liver preparations seem to be the most lucrative models with maximum feasibility. Cellular and subcellular systems are included in these models, and they can equally contain HepG2 and HuH7 hepatic cell lines (developed from liver tumors and preserved hepatocytes) [74,75,76]. The primary functional cells of the liver, the hepatocytes, have historically been challenging to culture ex vivo. Various complementary in vitro liver models have been introduced to overcome this difficulty. These models are classically categorized into 2D and 3D models. At the same time, none are simple and effective for predicting all hepatic functions (specifically, the clearance of chemicals).

Even though 2D models are flexible, affordable, and valuable for studies that require large numbers of cells, most cell lines do not have normal liver-specific functions, including those relevant to toxicology. They are genetically abnormal and do not adequately reproduce hepatocyte biology. Meanwhile, 3D cultures offer cell–cell and cell–extracellular matrix interactions, though these methods are often more challenging to translate into high-throughput tactics. Primary hepatocytes in 3D modeling can form spheroids, prolonging the maintenance of hepatic phenotype and function. The ability to transiently proliferate and self-organize is a well-known ability of hepatic cells, and it has also been taken advantage of in forming liver organoids. Organoid models have been developed from various hepatic cell types, and all exhibit various degrees of similarity to human hepatocytes [66,77,78]. Hepatocytes with or without non-parenchymal cells can be spatially patterned in 3D, using, for instance, soft lithography. Combining 3D printing technologies with cytocompatible biological “inks” enables engineers to bioprint tissue models, incorporating parenchymal and stromal cells in spatially patterned arrangements. Unfortunately, such “futuristic” models are challenging to make and maintain.

Since in vivo quetiapine metabolism pathways generate well-defined metabolite derivatives, this drug was used to explore the consistency of the in vitro metabolic model. Out of many emerging preclinical human-relevant in vitro models used to evaluate toxic injury to the liver, in silico modeling has shown good potential in terms of its affordability and easy maintenance [68]. Mathematical modeling, referred to as physiologically based pharmacokinetic (PBPK) modeling, is basically an in silico technique where mathematical modeling is used to inform and optimize the design in, for instance, forensic toxicology [79]. In the same context, forensic toxicologists should benefit from the estimated time course concentrations [80]. Reported human blood concentrations of quetiapine were considered in the context of the environment that includes the receptor (gut), metabolizing agent (liver), and central compartments with blood-to-plasma concentration ratios (Rb) and liver-to-plasma concentration ratios (Kp,h) [81,82]. Alternatively, some other compounds may be estimated using in silico tools. All of these are important clinical parameters for calculating pharmacokinetic (PK) properties [83]. In recent years, there has been significant progress in developing liver-emulating technologies, including liver-on-a-chip. Biochemical and metabolic information is chip-generated [84]. However, this advanced and highly sensitive technology is still in its infancy, as methodologies, procedures, and standards render the obtained data difficult to handle in the grossing room or in medicolegal settings in general [85].

### 4.5. Blood

**Figure 5:**
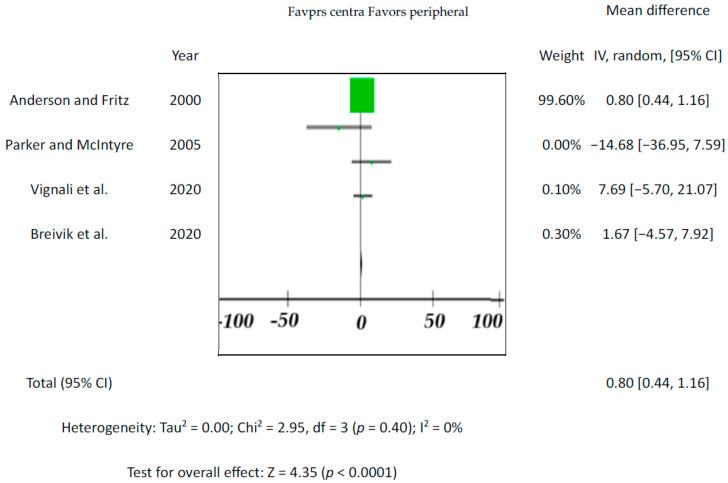
Forest plot of comparison of different blood matrices. For four studies that considered central and peripheral blood, pooled variance (Sp2) was calculated at 245.89 ng/mL with a pooled standard deviation of ±15.68 [18,44,45,50].

Since the drug’s blood level is the one that affects the individual, blood is the most important tissue for toxicological analysis. This accounts for central (e.g., heart) and peripheral (e.g., femoral) blood. Although there are cases where peripheral vs. central blood concentrations differed significantly, none of the five included studies showed significant differences between the endpoints (*p*-values varied from 0.18 to 0.59; for overall effect see Figure 5). The results from previous studies indicate that drug concentrations in the central blood are generally higher than in the peripheral blood [86].

### 4.6. Brain Tissue

Several studies considered brain tissue’s quetiapine, and in the study of Breivik et al. this concentration correlated moderately (positive correlation) with that in the peripheral blood (r = 0.5); unfortunately, the linear regression model was below the level of statistical significance [44]. Skov et al. even claim brain concentrations are about four times those in the blood [87] (see Table 4 containing data reviewed here).

### 4.7. Skeletal Muscle

Breivik et al. even concluded that, in the absence of blood, skeletal muscle may be treated as a preferred matrix for quetiapine concentrations since its concentration in skeletal muscle correlated well with that in peripheral blood. Hopenwasser et al.’s related data indicate a strong positive correlation between blood and skeletal muscle quetiapine concentrations. Their study participants showed r = 0.98 in a linear model with R^2^ = 0.97. Unfortunately, the *p*-value was, likewise, inappropriate at 0.07 [51]. A strong positive correlation (r = 0.92) was obtained in a linear model with a *p*-value of 0.98. The same was true in the cohort of Vignali et al. in which a strong correlation was obtained for a poor model (r = 0.80, R2 = 0.63, *p*-value = 0.51) [44,45]. Nevertheless, quetiapine’s implication in the metabolism of lipids in the skeletal muscle is visible in lipidomics [88].

In conclusion, Burghardt et al.’s findings suggest that atypical antipsychotics change the lipid profiles of human skeletal muscle, so the role of that tissue in quetiapine metabolism should be assessed in the future [62,88]. Precisely because of this, it should be no surprise that Breivik et al. (b) validated the method for determining quetiapine in postmortem skeletal tissue [89].

## 5. Other Matrices

The blood, the brain, the liver, and the muscle tissue were all used as preferred matrices in those five studies of interest to this review. Of 238 samples assessed, 59 (24.79%) were liver tissue. Blood, as a primary tissue of toxicological interest, whether central or peripheral, was considered in 54/238 (22.59%) and 43/238 (18.07%) cases, respectively. Brain and skeletal muscle were both bordering on 10% of cases. The brain was assessed in 25/238 (10.5%), and the skeletal muscle in 247/38 (10.1%).

**Figure 6:**
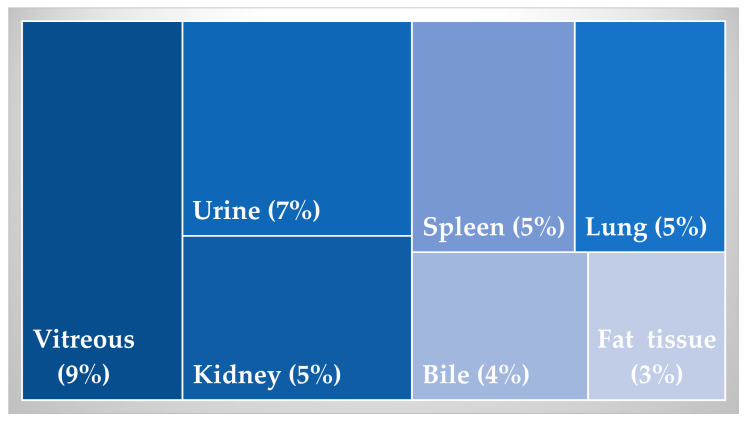
Less frequently used matrices.

Other less frequently used matrices are given in Figure 6.

Pathologists’ choices seem unfortunate since bile and vitreous are traditionally preferred matrices in forensic toxicology [90]. More so considering moderate positive correlations of femoral blood’s quetiapine and the concentrations of quetiapine in vitreous or bile. However, peripheral blood’s quetiapine and its concentrations in vitreous correlated (though weakly) in the study of (r = 0.32, R^2^ = 0.11, *p*-value = 0.04) [18]. Vignali et al. correlated bile with peripheral blood more straightforwardly (r = 0.52, R^2^ = 0.28, *p*-value = 0.01) [45].

Lastly, quetiapine concentrations in hair segments have been assessed. Such an assessment is a step forward in therapeutic drug monitoring. Not counting the practical significance of this endpoint in forensic toxicology. Unfortunately, none of the five studies considered included hair concentrations of quetiapine, so more detailed calculations are unavailable. For the completeness of this review, note that several studies have been published over the years on antipsychotics in hair, and quetiapine is not an exception. Studies also report quetiapine concentrations in nails [91,92,93,94]. Nevertheless, nails should be preferred as a relevant matrix since they retain certain substances more likely to have concentrations than hair [95,96].

## 6. Conclusions

When blood is not available, the analysis of other tissues can provide important information that helps diagnose potential intoxication with quetiapine. The search for an adequate alternative endpoint seems rational, considering the increasing trend of quetiapine misuse and overdoses.

The relatively high concentrations of quetiapine in the liver tissue, and the modest (if any) statistical significance when correlating other endpoints with blood, cast a suspicion on any straightforward recommendation for selecting a relevant matrix. Further investigations and the integration of results obtained in silico and in vitro are needed to improve routine forensic toxicology. Recent endeavors where hair or nails were used as surrogate endpoints point out the advantages of keratin matrices that are much more resistant to post-mortem decomposition than other biological samples. Even though the evidence on the feasibility of keratinized matrices in this regard is missing, these reports could answer this paper’s query.

## Figures and Tables

**Table 1:** Characteristics of the included studies.

Study	Year	Participants	Method	Interventions	Correction of the Measurement Units
Anderson and Fritz [50]	2000	7	Experimental case series	Postmortem toxicology	no
Hopenwasser et al. [51]	2004	8	Experimental case series	Postmortem toxicology	no
Parker and McIntyre [18]	2005	21	Experimental case series	Postmortem toxicology	no
Vignali et al. [45]	2020	13	Experimental case series	Postmortem toxicology	yes
Breivik et al. [44]	2020	14	Experimental case series	Postmortem toxicology	no

**Table 2:** Table of correlations and a goodness of fit for quetiapine concentrations in liver tissue vs. bile or stomach content.

Study	Year	Pearson Correlation Coefficient (r)	R2 (Goodness of Fit)	*p*-Value	Matrix
Anderson and Fritz [50]	2000	0.21	0.04	0.22	Bile
Anderson and Fritz [50]	2000	0.51	0.26	0.02	Gastric content
Hopenwasser et al. [51]	2004	0.99	0.97	0.19	Bile
Hopenwasser et al. [51]	2004	−0.33	0.11	0.23	Gastric content
Parker and McIntyre [18]	2005	−0.15	0.02	0.04	Gastric content
Vignali et al. [45]	2020	0.52	0.28	0.0005	Bile

**Table 3:** Correlation and goodness of fit for peripheral blood and liver tissue.

Study	Year	Pearson Correlation Coefficient (r)	R^2^ (Goodness of Fit)	*p*-Value
Anderson and Fritz [50]	2000	0.82	0.68	0.23
Hopenwasser et al. [51]	2004	−0.28	0.08	0.94
Parker and McIntyre [18]	2005	0.37	0.14	0.04
Breivik et al. [44]	2020	0.82	0.66	0.27
Vignali et al. [45]	2020	−0.26	0.07	0.25

**Table 4:** Table of correlations and goodness of fit for quetiapine concentrations in peripheral blood vs. brain tissue.

Study	Year	Pearson Correlation Coefficient (r)	R2 (Goodness of Fit)	*p*-Value
Hopenwasser et al. [51]	2004	Only two samples	0.19	
Breivik et al. [44]	2020	0.50	0.25	0.24
Vignali et al. [45]	2020	0.05	0.002	0.25

## Data Availability

Available on request.
